# Effects of GLP-1 receptor agonist therapy on resolution of steatohepatitis in non-alcoholic fatty liver disease: a systematic review and meta-analysis

**DOI:** 10.1093/jcag/gwae057

**Published:** 2025-01-29

**Authors:** Kathryn J Potter, Jackie Phinney, Tasha Kulai, Vicki Munro

**Affiliations:** Department of Pediatrics, Cumming School of Medicine, University of Calgary, Calgary, AB, T2N 4Z5, Canada; Dalhousie University, Dalhousie Medicine New Brunswick, Saint John, NB, E2L 4L5, Canada; Division of Digestive Care and Endoscopy, Dalhousie University, Halifax, NS, B3H 2Y9, Canada; Division of Endocrinology, Dalhousie University, Halifax, NS, B3H 2Y9, Canada

**Keywords:** NAFLD, GLP-1, Steatohepatitis

## Abstract

**Background:**

Non-alcoholic fatty liver disease (NAFLD) is common, can progress to cirrhosis and hepatic decompensation, and has no approved medical therapy in Canada.

**Objective:**

We conducted a systematic review on whether glucagon-like peptide-1 receptor agonists (GLP-1RA) improve non-alcoholic steatohepatitis (NASH) compared to standard care in NAFLD.

**Methods:**

We searched Medline Ovid, EMBASE(Elsevier), Cochrane CENTRAL, Clinical Trials.gov, and the World Health Organization International Clinical Trials Registry Platform in November 2023 for randomized controlled trials. Inclusion criteria included patients ≥13 years with NAFLD receiving GLP-1RA for ≥6 months compared to standard care/placebo. Cochrane risk-of-bias 2.0 tool was used for each outcome. After screening results in duplicate, we performed meta-analysis and reported odds ratios (OR) for dichotomous and mean difference of change score for continuous outcomes.

**Results:**

Six studies with 478 patients met inclusion criteria; 3 studies reported on the primary endpoint resolution of NASH. GLP-1RA likely leads to resolution of NASH (OR 4.45 (95% CI 1.92, 10.3)) and reduction in liver steatosis on imaging (–5.09% (95% CI −7.49, −2.69), but little to no reduction in liver stiffness on imaging (mean difference –0.17 kPa (95% CI −0.34, 0)).

**Interpretation:**

Treatment with GLP-1RA in NAFLD patients for ≥6 months can probably lead to improvement in NASH on liver biopsy and reduce liver steatosis on imaging. Whether improvements in steatosis on biopsy or imaging results in clinically significant outcomes need to be elucidated as the effects of GLP-1RA on liver fibrosis are unclear; larger ongoing trials may provide more definitive answers.

**Protocol Registration:** PROSPERO–CRD42023472186.

Non-alcoholic fatty liver disease (NAFLD), is prevalent in ~22% of the Canadian population^1^ and is the second most common cause of liver transplantation.^2^ NAFLD encompasses a spectrum of diseases, including intrahepatic steatosis with minimal inflammation and non-alcoholic steatohepatitis (NASH) with or without fibrosis. Patients with NASH and ≥ stage 2 fibrosis have a higher likelihood of developing cirrhosis, hepatic decompensation, hepatocellular carcinoma, and cirrhosis-related death.^3^ Liver biopsy is the gold standard to diagnose NASH^3^ but is only performed in cases of diagnostic uncertainty. Non-invasive imaging techniques are a less invasive and less costly alternative; vibration-controlled transient elastography (VCTE), and magnetic resonance imaging with proton density fat fraction (MRI-PDFF) can be used to identify steatosis, while VCTE and magnetic resonance elastography (MRE) can be used to identify fibrosis.^3^ Both VCTE and MRE have high predictive value of liver-related outcomes.^4^ While MRE has shown excellent correlation to the gold standard biopsy,^5^ it is less easily accessible outside clinical trials. VCTE however can have more variable results, particularly in those with cirrhosis.^4^

As there are no approved medications in Canada, the mainstay of treatment for NAFLD is lifestyle interventions targeting loss of 7%-10% body weight^3^ but is achieved in <10% of patients.^2^ Glucagon-like-peptide receptor agonists (GLP-1RA) have emerged as a potential treatment. GLP-1RA is a modified incretin hormone that promotes satiety, potentiates insulin secretion, slows gut motility, and has anti-apoptotic and metabolic regulatory roles in many tissues.^6,7^ They are approved for treatment of type 2 diabetes (T2D) and obesity.^7,8^ GLP-1RA may directly or indirectly (by reduction of obesity and insulin resistance) protect against and/or treat NAFLD.^9^ In vitro studies suggest hepatocytes may express GLP-1 receptors and GLP-1RA directly reduce hepatic lipogenesis through AMPK signalling.^10^

Previous systematic reviews have evaluated GLP-1RA use in patients with obesity, T2D, and polycystic ovarian syndrome, demonstrating improvements in liver enzymes and steatosis on imaging in patients with or without confirmed NAFLD.^11–23^ Over time, with guidance from agency recommendations,^24,25^ randomized controlled trials (RCT) have accumulated in GLP-1RA treatment for NAFLD, using more standardized liver imaging and biopsy as primary endpoints. Given the new publications in recent years, we performed a systematic review and meta-analysis to determine whether GLP-1RA therapy improves hepatic steatosis compared to standard care or placebo in patients with NAFLD using RCTs only.

## Methods

### Search strategy and study selection

We adhered to the Preferred Reporting Items for Systematic Reviews and Meta-Analysis (PRISMA) guidelines^26^ and PRISMA-S.^27^ A comprehensive search strategy containing subject headings and keywords was developed and tested by a medical librarian (JP) using Medline (Ovid). This search strategy incorporated the updated RCT search filter in the Cochrane Handbook for Systematic Reviews of Interventions,^28^ and after undergoing peer-review using the PRESS checklist,^29^ was updated and translated to proper search syntax for each database. Medline (Ovid), EMBASE (Elsevier), Cochrane CENTRAL (Cochrane Library), Clinical Trials.gov (www.clinicaltrials.gov), and the World Health Organization (WHO) International Clinical Trials Registry Platform (ICTRP) (www.who.int/ictrp/search/en/) were searched on November 16, 2023. No date or language limits were applied and the detailed search strategies are described in Supplementary Appendix 1. The resulting database records were uploaded to Covidence^30^ with duplicates both automatically and manually removed. The ClinicalTrials.gov and WHO ICTRP results were exported to an Excel workbook and duplicate records were manually removed.

Our inclusion criteria focussed on RCTs with participants aged ≥13 years with clinical diagnosis of NAFLD confirmed by non-invasive imaging^2^ or liver biopsy treated with any GLP-1RA for ≥6 months compared to placebo or standard of care. Our search strategy included both NAFLD and metabolic-associated fatty liver disease (MAFLD), which was proposed as a new term in 2020.^31^ We did not search for the term metabolic-associated steatotic liver disease (MASLD), which was not officially implemented until after our search strategy was written and piloted. We excluded participants post-liver transplantation, dual GLP-1RA, and gastric inhibitory polypeptide (GIP) receptor agonist therapy or dual GLP-1RA and glucagon therapy, with prior exposure to GLP-1RA, and/or concurrent treatment with another active medical therapy while on GLP-1RA. The primary outcome was the resolution of steatohepatitis without worsening of fibrosis. Secondary outcomes included: change in steatosis by magnetic resonance spectroscopy, change in liver stiffness by MRE or VCTE, change in fibrosis stage on biopsy, change in liver enzymes (AST, ALT), change in body weight, change in BMI, and rates of serious adverse events. Two reviewers (KP, VM) independently screened abstracts and full-text articles using prespecified inclusion and exclusion criteria and resolved discrepancies by discussion and consensus.

### Data extraction and risk of bias

Two reviewers (VM, KP) independently extracted data using a pre-specified data abstraction form and then resolved differences by discussion and consensus. The risk of bias (ROB) was assessed using the Cochrane ROB 2.0 tool.^32^ Two reviewers (KP, VM) independently evaluated ROB and each outcome was judged to have “low risk of bias” if all domains were low risk, “some concerns of bias” if there were concerns in at least 1 domain, and “high risk of bias”, if one or more domains were high risk or if there were some concerns in multiple domains.

### Data analysis

Odds ratios and 95% CI were calculated from the number of events or participants in each group for dichotomous outcomes. Continuous outcomes were reported as the within-group change from baseline score with 95% CI and obtained the mean difference for the between study effect estimates. When a standard deviation (SD) for a change score was not available, it was estimated by multiple imputations using a correlation coefficient calculated from the SD in baseline and change measurement in other studies with available data.^33^ Data synthesis was performed using Review Manager (Version 6.7.0, The Cochrane Collaboration, revman.cochrane.org). The summary effect for continuous variables was calculated using inverse variance and a random effects model. The summary effect for dichotomous variables was calculated using the Mantel–Haenszel method and a random effects model. Statistical heterogeneity analysis across trials was assessed by *χ*² test (<0.10 considered significant) and the *I*² statistic.

### Subgroup and sensitivity analysis

Subgroup analysis was performed to investigate the sources of potential heterogeneity in the type of control (placebo vs stringent lifestyle modifications) and for the scale used to assess for liver fibrosis. Sensitivity analysis was performed to determine whether the exclusion of studies of high risk of bias changed the treatment effect size and statistical significance of the meta-analysis.

### Certainty of evidence

The Grading of Recommendations Assessment, Development, and Evaluation (GRADE) approach^34^ was used to assess the certainty of evidence using the GRADEpro Guideline Development Tool.^35^

## Results

**Figure 1.**
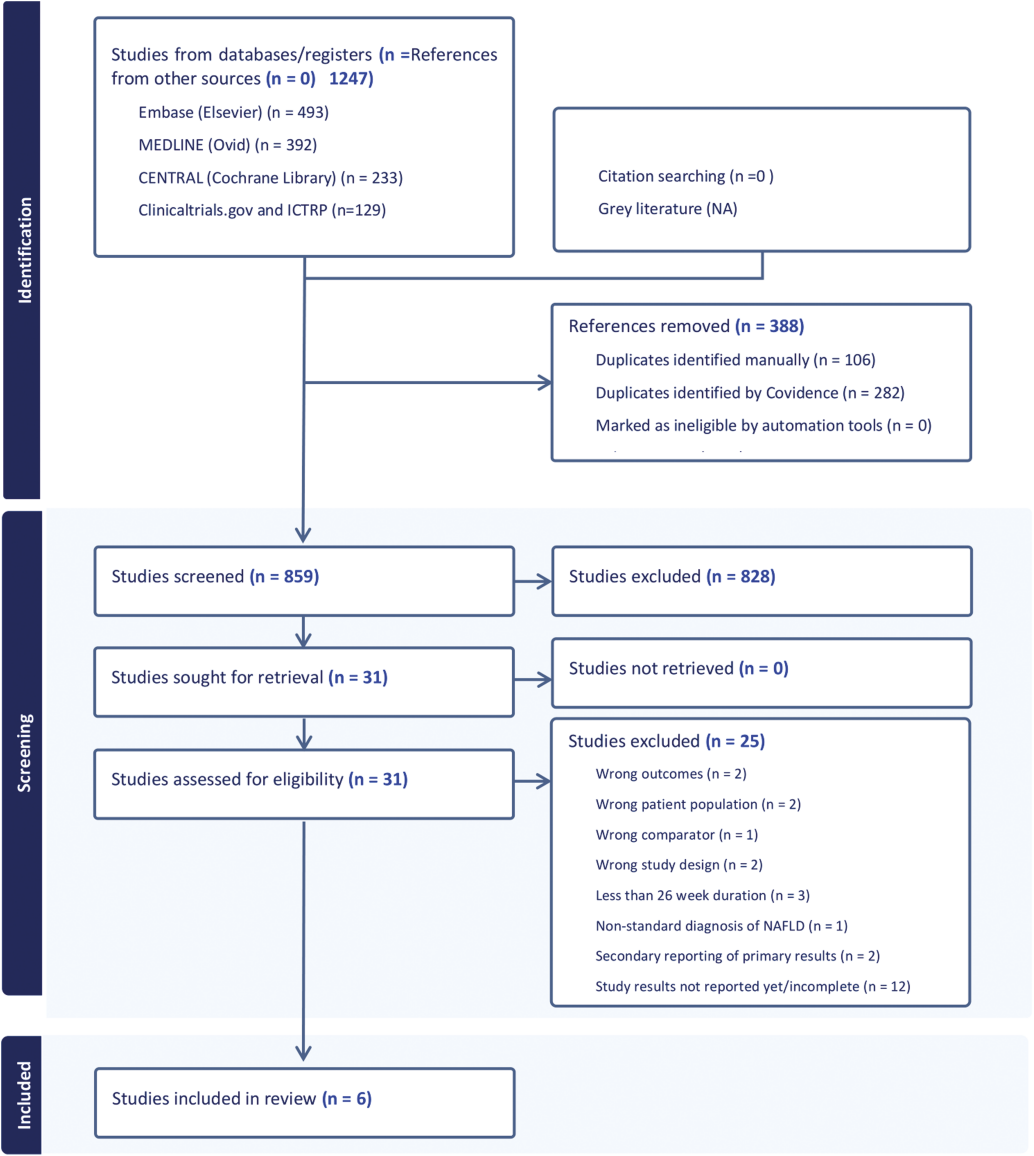
PRISMA flow diagram.

The PRISMA flow diagram (Figure 1) describes our review process. After the retrieval of 1247 studies and the removal of 388 duplicates, 859 studies were considered. Of these, 31 underwent full-text review, with 25 studies excluded. Formal review, risk of bias assessment, and data extraction were performed for the remaining 6 studies.^36–41^

### Study characteristics

Characteristics of the included studies are summarized in Table 1. Studies were published between 2016 and 2023 and enrolled a total of 631 participants. Newsome et al.^41^ included 3 doses of semaglutide (0.1, 0.2, and 0.4 mg); we excluded the 2 lower doses of semaglutide. Guo et al.^40^ had 3 arms: placebo (30), GLP-1RA (31), and insulin glargine (30); we excluded the insulin glargine arm. We therefore included 443 participants in the analysis.

**Table 1.** Baseline characteristics of included studies.

Author, year of publication	Location	No. patients	Female (%)	Mean age (years)	Method of NAFLD diagnosis	Patients with T2DM (%)	Intervention and dose	Control	Treatment duration (weeks)	Outcome evaluation
Armstrong, 2016	UK	52	40	51	Biopsy	35	Liraglutide 1.8 mg daily	Placebo	48	Liver biopsy
Khoo, 2019	Singapore	30	10	40.7	MRI-PDFF	Not specified	Liraglutide 3.0 mg daily	Lifestyle (exercise and caloric restriction)	26	MRE, MRI-PDFF
Guo, 2020	China	96	44	52.6	Imaging	100	Liraglutide 1.8 mg daily	Placebo	26	H-MRS
Flint, 2021	Germany	67	29.9	60	MRE and MRI-PDFF	73	Semaglutide 0.4 mg daily	Placebo	72	MRE
Newsome, 2021	16 countries (Europe, Asia, North America and Australia)	320	60.6	55	Biopsy	62	Semaglutide 0.4 mg daily	Placebo	72	Liver biopsy
Loomba, 2023	Multiple European countries and USA	71	69	59.5	Biopsy	75	Semaglutide 2.4 mg weekly	Placebo	48	Liver biopsy

**Figure 2.**
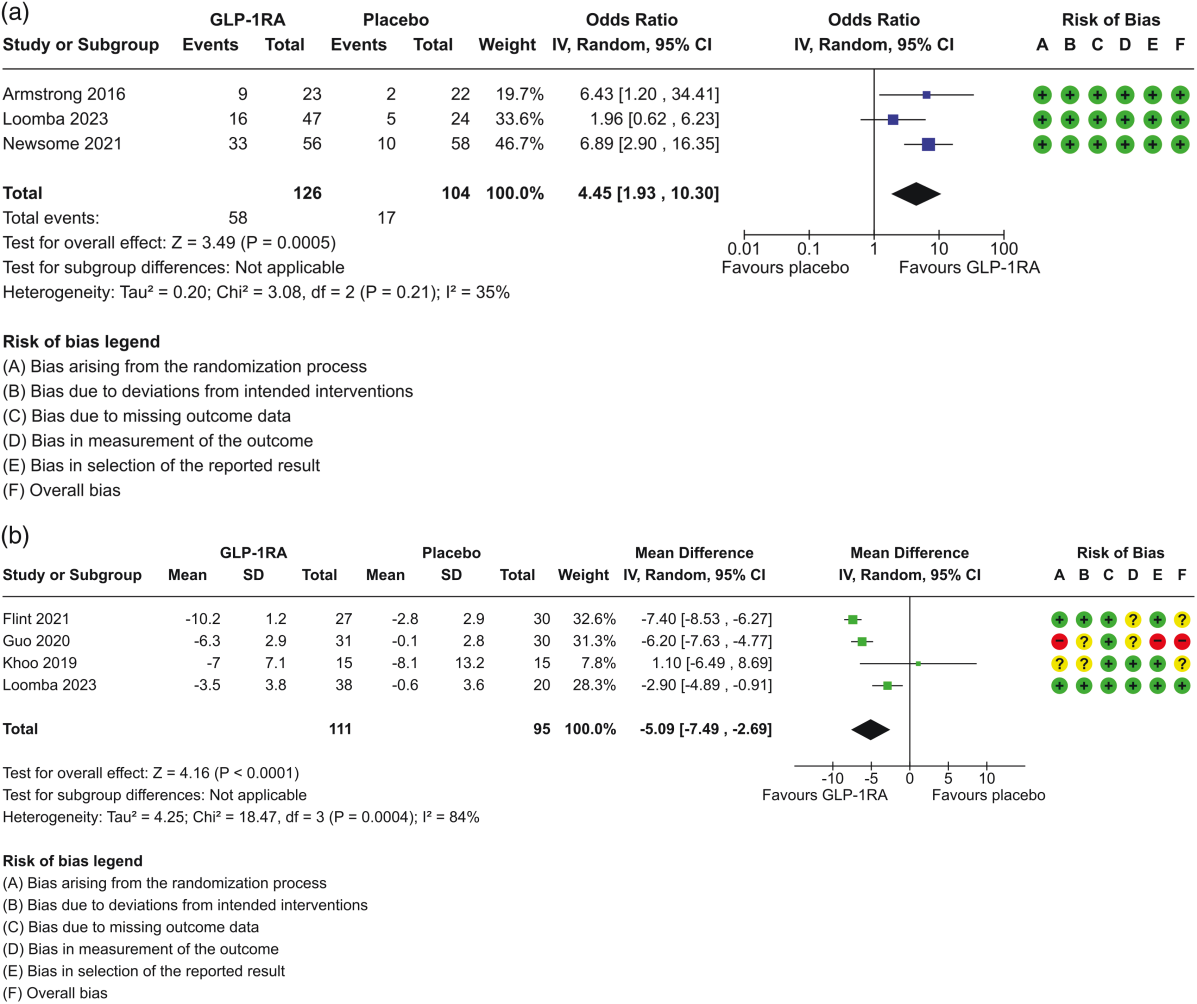
Forest plot to assess the effect of GLP-1RA measures of hepatic steatosis by (a) resolution of steatohepatitis on liver biopsy and (b) reduction in hepatic steatosis as measured by MRI-PDFF(%) over 26–72 weeks. *For the risk of bias assessment, a circle with a “+” sign symbolizes “low risk of bias,” a circle with a “?” symbolizes “some concerns of potential bias,” and a circle with a “-“ symbolizes “high risk of bias.”

**Figure 3.**
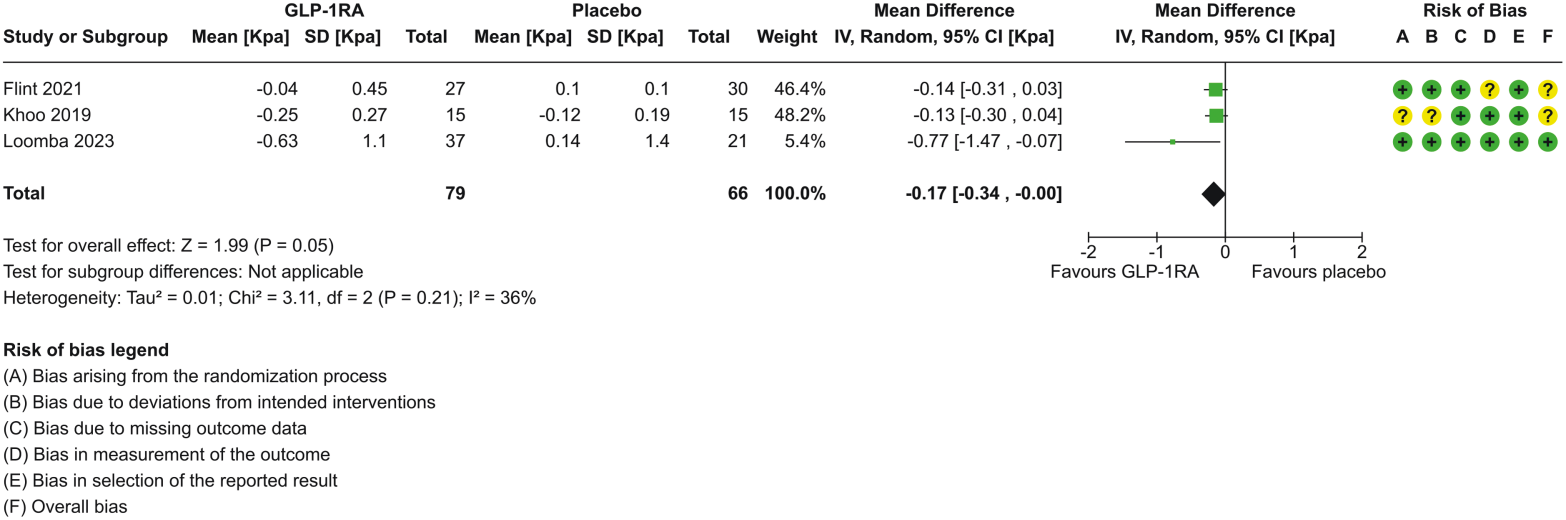
Forest plot to assess the effect of GLP-1RA on the change fibrosis by MR-elastography over 26–72 weeks. *For the risk of bias assessment, a circle with a “+” sign symbolizes “low risk of bias,” a circle with a “?” symbolizes “some concerns of potential bias,” and a circle with a “-“ symbolizes “high risk of bias.”

**Figure 4.**
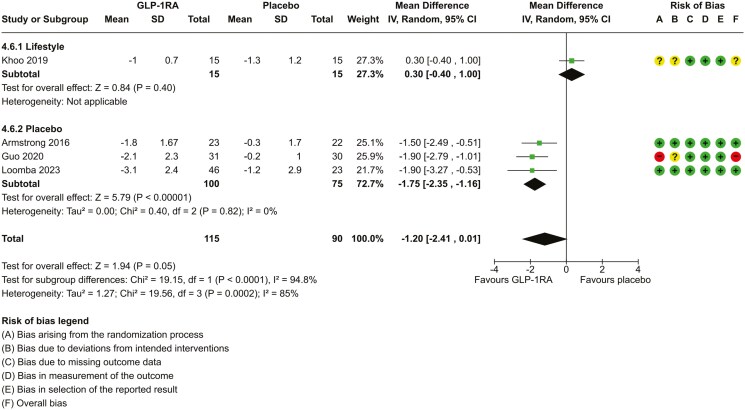

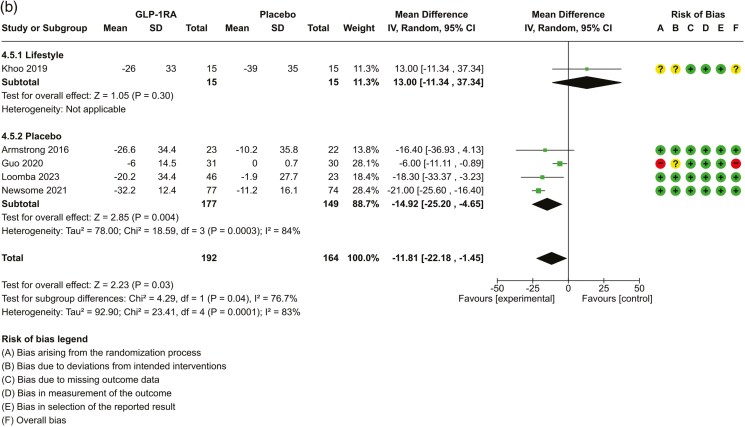

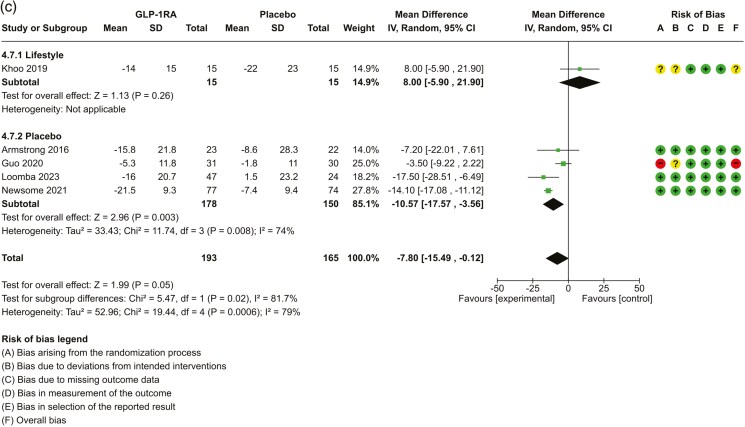
Forest plot to assess the effect of GLP-1RA (a) body mass index (BMI) and liver enzymes; (b) alanine aminotransferase, or ALT; (c) aspartate aminotransferase, or AST over 26–72 weeks. *For the risk of bias assessment, a circle with a “+” sign symbolizes “low risk of bias,” a circle with a “?” symbolizes “some concerns of potential bias,” and a circle with a “-” symbolizes “high risk of bias.”

Five studies compared GLP-1RA to a placebo group^36–38,40–42^ while one^39^ compared to a supervised dietary restriction and exercise regimen. For the treatment group, varying GLP-1RA and doses were used: liraglutide 1.8 mg daily (57 patients), liraglutide 3 mg daily (15 patients), semaglutide 0.4 mg daily (116 patients), and semaglutide 2.4 mg weekly (47 patients). Treatment duration ranged from 26 to 72 weeks. Two studies enrolled Asian participants only, the remainder had predominantly white participants. Concomitant T2D varied across studies, ranging from 35% to 100%. ROB assessments for each outcome are presented in Figures 2-4 (details in Supplemental Table 1). A summary of the findings table provides details on GRADE rating (Table 2).

**Table 2.** Summary of findings table.

GLP-1 receptor agonist compared to standard of care in NAFLD						
Patient or population: NAFLD Intervention: GLP-1 receptor agonist Comparison: standard of care						
Outcomes	Anticipated absolute effects* (95% CI)		Relative effect (95% CI)	No. of participants (studies)	Certainty of the evidence (GRADE)	Comments
	Risk with standard of care	Risk with GLP-1 receptor agonist				
Resolution of NASH assessed with: liver biopsy follow-up: range 55 weeks to 72 weeks	16 per 100	47 per 100 (27 to 67)	OR 4.45 (1.93 to 10.30)	230 (3 RCTs)	⨁⨁⨁◯ Moderate^a^^,^^b^^,^^c^^,^^d^	GLP-1RA probably increase resolution of NASH but the certainty of the evidence is moderate.
% change in hepatic steatosis assessed with: MRI-PDFF (%) follow-up: range 26 weeks to 72 weeks	The mean % change in hepatic steatosis ranged from **−8.1 to −0.1** %	MD **5.63% lower** (7.96 lower to 3.29 lower)	-	176 (3 RCTs)	⨁◯◯◯ Very low^d^^,^^e^^,^^f^^,^^g^	GLP-1RA may reduce hepatic steatosis assessed by MRI-PDFF but the certainty of the evidence is very low.
Change in hepatic fibrosis assessed with: MRE follow-up: range 52 weeks to 72 weeks	The mean change in hepatic fibrosis ranged from −0.12 to 0.14 kPa	MD 0.17 kPa lower (0.34 lower to 0)	-	145 (3 RCTs)	⨁⨁◯◯ Low^d,^^h^^,^^i^^,^^j^	GLP-1RA have little to no effect on hepatic fibrosis assessed by MR-elastography, though the certainty of the evidence is low.
Change in BMI assessed with: kg/m2 follow-up: range 26 weeks to 60 weeks	The mean change in BMI ranged from −1.3 to −0.2 kg/m^2^	MD 1.2 kg/m2 lower (2.41 lower to 0.01 higher)	-	205 (4 RCTs)	⨁⨁◯◯ Low^d^^,^^k^^,^^l^^,^^m^	GLP-1RA may reduce BMI, but the certainty of the evidence is low.
Change in ALT assessed with: Units/L follow-up: range 26 weeks to 72 weeks	The mean change in ALT ranged from −39 to 0 U/L	MD 11.81 U/L lower (22.18 lower to 1.45 lower)	–	356 (5 RCTs)	⨁◯◯◯ Very low^d^^,^^k^^,^^n^^,^^o^	GLP-1RA may reduce ALT levels, but the certainty of the evidence is very low.
Change in AST assessed with: Units/L follow-up: range 26 weeks to 72 weeks	The mean change in AST ranged from −22 to 1.5 U/L	MD 7.80 U/L lower (15.49 lower to 0.12 lower)	–	358 (5 RCTs)	⨁◯◯◯ Very low^d^^,^^k^^,^^p^^,^^q^	GLP-1RA may reduce AST levels, but the certainty of the evidence is very low.

*The risk in the intervention group (and its 95% confidence interval) is based on the assumed risk in the comparison group and the relative effect of the intervention (and its 95% CI).

CI: confidence interval; MD: mean difference; OR: odds ratio

GRADE Working Group grades of evidence

High certainty: we are very confident that the true effect lies close to that of the estimate of the effect.

Moderate certainty: we are moderately confident in the effect estimate: the true effect is likely to be close to the estimate of the effect, but there is a possibility that it is substantially different.

Low certainty: our confidence in the effect estimate is limited: the true effect may be substantially different from the estimate of the effect.

Very low certainty: we have very little confidence in the effect estimate: the true effect is likely to be substantially different from the estimate of effect.

Explanations.

^a^Low risk of bias across all studies.

^b^Low level of heterogeneity between studies suggested by a non-significant *χ*² *P*-value for heterogeneity, an *I*^2^ value of 35%, and visual inspection of the Forest plot.

^c^Insufficient participants to meet the optimal information size (OIS) of 1562 calculated using a control group event rate of 0.16, alpha of 0.05, beta of 0.2, and a chosen relative risk reduction (RRR) of 30%.

^d^Majority of studies funded by industry.

^e^Lack of allocation concealment in an unblinded study and some concerns in 2 other studies.

^f^High level of heterogeneity, with a *P*-value for heterogeneity of 0.004 and an *I*^2^ value of 84%.

^g^Insufficient participants to meet the optimal sample size. Using a mean steatosis of 9% in an average NAFLD population (add ref), and a suggested minimally important difference of 30% reduction in liver fat (Caussy), we set a minimally important difference threshold for absolute change detection of 2.7%. Using an alpha of 0.05 and a beta of 0.2, along with a SD of 7.1 from an included trial, the optimal sample size was calculated as 256 which was not met.

^h^Some concerns in multiple domains of 2 of 3 studies.

^i^Non-significant *P* value for heterogeneity and the *I*^2^ value of 36%.

^j^We set a minimally important difference threshold for absolute change detection of 0.3 kPa. Using an alpha of 0.05 and a beta of 0.2, along with a SD of 0.97 from an included trial, the optimal sample size was calculated as 328 which was not met.

^k^One study with a high risk of concerns in one domain and some in another and a second study with some concerns in multiple domains.

^l^Subgroup analysis identified the cause of heterogeneity and the *P*-value of 0.82 and *I*^2^ of 0% for the 3 studies in the placebo group showed that there was no heterogeneity between the studies in this subgroup. There was no heterogeneity for the single paper in the lifestyle subgroup.

^m^Insufficient participants to meet the optimal sample size. Decrease in BMI of >1 appears to be associated with lower RR of NAFLD and setting a minimally important difference of 1 with an SD 5.9, the optimal information size of 1094 was not met.

^n^High level of heterogeneity between all studies overall as well as the studies in the placebo group in subgroup analysis (*P* value for heterogeneity 0.0003, I2 84%) and heterogeneity not explained by subgroup analysis.

^o^Insufficient participants to meet the optimal sample size. A minimally important difference was set based on previous studies showing dietary changes alone can elicit a mean difference of −4.48; given an alpha of 0.05 and beta of 0.20 with SD 32, the optimal sample size of 1024 was not met.

^p^High level of heterogeneity between all studies overall as well as the studies in the placebo group in subgroup analysis (*P* value for heterogeneity 0.008, *I*^2^ 74%) and heterogeneity not explained by subgroup analysis.

^q^Insufficient participants to meet the optimal sample size. A minimally important difference was set based on previous studies showing lifestyle changes alone can elicit a mean difference of −7.33 U/L; given an alpha of 0.05 and beta of 0.20 with SD 23 (Khoo), the optimal sample size of 310 was not met.

### Effect of GLP-1RA on resolution of MASH and reduction of hepatic steatosis

#### Resolution of MASH

Three studies including 230 participants reported on the resolution of MASH without worsening fibrosis on liver biopsy as an outcome. All 3 studies were deemed low ROB. The Forest plot is shown in Figure 2A. The odds ratio (OR) was 4.45 (95% CI 1.92, 10.3) in favour of GLP-1RA. Although there was only moderate overlap of confidence intervals (CI), a *χ*^2^*P*-value of 0.21 and an *I*^2^ value of 35% suggested a low level of heterogeneity between studies. We rated moderate certainty in the evidence (Table 2).

#### Improvement in Steatosis

Four studies including 206 participants assessed the effect of GLP-1RA on the change in the proportion of steatosis using non-invasive imaging (MRI-PDFF). The mean steatosis at baseline was 19.1% (SD 7.4). The Forest plot is shown in Figure 2B. Two studies^36,38^ reported results as baseline and post-intervention steatosis, and the change score SD was calculated by imputation from the other 2 studies.^39,40^ The pooled mean difference in the change in liver steatosis was –5.09% (95% CI, −7.49, −2.69). The lack of overlap of all CI, a *χ*^2^*P*-value of 0.0004, and *I*^2^ of 84% demonstrated a high level of heterogeneity. We rated very low certainty in the evidence (Table 2). In a sensitivity analysis in which we removed one study with high ROB, the pooled mean difference in the change in liver steatosis was –4.09% (95% CI, −8.27, 0.01) (Supplementary Figure 1). A *χ*^2^*P*-value of 0.06, and *I*^2^ of 89% suggested that heterogeneity between studies was not explained by the removal of the high ROB study. Overall, GLP-1RA had a range of reduction in steatosis of 22%-56% from baseline.

### Effect of GLP-1RA on hepatic fibrosis

#### Improvement in hepatic fibrosis on liver biopsy

Three studies including 222 participants, assessed improvement in fibrosis on liver biopsy, 2 using the Kleiner fibrosis scale (71 participants) and 1 using the Ishak fibrosis scale (151 participants). There were insufficient studies using either fibrosis scale to perform meta-analysis. In the 2 studies using the Kleiner fibrosis scale, the OR of improvement in the fibrosis stage was not statistically significant at 1.42 (95% CI, 0.62, 3.28)^41^ and 1.54 (95% CI, 0.72, 3.30).^37^ The study using the Ishak fibrosis scale showed discrepant findings with fibrosis improvement not statistically significant in favour of placebo, OR 0.28 (95% CI, 0.06-1.24).^36^

#### Improvement in hepatic fibrosis on imaging

Four studies reported change in fibrosis using non-invasive imaging—3 using MRE (145 participants) and 1 using VCTE (162 participants). While the units (kPa) are the same, the scales of MRE and VCTE are different and cannot be combined for meta-analysis.^3^ Meta-analysis was performed using continuous data from the 3 studies using MRE (Figure 3). The SD for change in hepatic fibrosis by MRE was not available for 2 studies^36,38^ and was derived by single imputation using a correlation coefficient derived from the third study.^39^ The pooled mean difference was –0.17 kPa (95% CI, −0.34, 0), favouring improvement in liver stiffness in the GLP-1RA treatment. There was limited overlap of CI, a *χ*^2^*P*-value of 0.21, and an *I*^2^ value of 36%, suggesting low heterogeneity between studies. We rated low certainty in the evidence (Table 2). The range of improvement in liver stiffness was 9%-13% from baseline in those treated with GLP-1RA.

### Effect of GLP-1RA on body weight and BMI

Data was available on change in weight in 5 studies (Supplementary Figure 2) and BMI for 4 studies (Figure 4A). The overall mean difference of treatment effect for weight was –4.77 kg (95% CI, −1.51, −2.51). There was high heterogeneity between studies (lack of overlap of CI, *χ*^2^*P*-value of <0.00001, *I*^2^ = 89%).

The overall mean difference of treatment effect for BMI was –1.2 kg/m^2^ (95% CI, −2.41, 0.01) in favour of GLP-1RA. There was high heterogeneity between studies (lack of overlap of CI, *χ*^2^*P*-value = <0.00001, *I*^2^ = 85%). We performed a subgroup analysis according to the type of comparator (lifestyle vs placebo). In the lifestyle study, the treatment effect was 0.3 (95% CI, −0.4, 1.0) in favour of lifestyle intervention. In the placebo comparator groups, the mean difference was –1.75 (95% CI, −2.35, −1.16) in favour of GLP-1RA. There was minimal heterogeneity between these 3 studies (χ^2^*P* value = 0.82, *I*^2^ = 0%). There was a significant difference between subgroup effects (*P* < 0.0001). We rated low certainty in the evidence (Table 2).

### Effect of GLP-1RA on liver enzymes

Data was available to assess change in ALT (Figure 4B) and AST (Figure 4C) for 5 studies, of which one used lifestyle interventions for a comparator group and 4 used placebo. Change score SDs for both ALT and AST were not available for 2 studies^36,41^ and were derived from multiple imputations of other studies.^38,40^ The overall mean difference in ALT was –11.81 U/L (95% CI −22.18, −1.45, *χ*^2^*P*-value of <0.0001, *I*^2^ = 83%). The overall effect for the mean difference in AST for all 5 studies was –7.8 U/L (95% CI, −15.49, −0.12). There was high heterogeneity between studies (lack of overlap of CI, χ^2^*P* value = 0.0006, *I*^2^ = 79%). We rated low certainty in the evidence for both AST and ALT (Table 2). There was a range of 18%-60% reduction in ALT and 18%-49% reduction in AST from baseline in those treated with GLP-1RA.

### Effect of GLP-1RA on serious adverse events

All studies reported serious adverse events with a total of 442 participants. The odds ratio for serious adverse events was 1.45 (95% CI, 0.73, 2.89; Supplementary Figure 3).

## Interpretation

Our results show that GLP-1RA therapy likely increases the odds of resolution of MASH by 4.45 times the odds of standard care. GLP-1RA may reduce liver steatosis on imaging but the evidence is very uncertain. There was insufficient evidence to assess the effect of GLP-1RA on fibrosis by biopsy. GLP-1RA may result in a slight improvement in fibrosis on imaging. With regard to other secondary outcomes, GLP-1RA may result in a reduction in BMI as well as AST and ALT levels, but the evidence is very uncertain.

Previous reviews of GLP-1RA therapy have reported improvement in liver fat content^11–15,19-^ on non-invasive testing (in those with or without confirmation of NAFLD). It is unclear if this leads to protection from the development of NAFLD or future sequelae of chronic liver disease. In those with an established diagnosis of NAFLD, GLP-1RA also appears to lead to improvement in liver fat content.^11,19,23^ Our results are in line with recent reviews, including one focused on semaglutide alone,^23^ suggesting a broader class effect to GLP-1RA treatment. The clinical significance of the effect may be small, as a >30% relative decrease in MRI-PDFF liver fat is associated with histologic response,^42^ and with an absolute mean pooled difference of 5.09%, the relative difference was 26.6%.

We attempted to assess biopsy-proven improvement in liver fibrosis with GLP-1RA therapy, but there was insufficient data. We did identify several trials^43–46^ that are ongoing with longer treatment durations and using histological outcomes which will hopefully provide more definitive answers. Using a surrogate measure by MRE, GLP-1RA treatment for up to 26 weeks leads to little to no reduction in liver fibrosis. While previous studies have shown potential improvement in liver fibrosis biomarkers^21^ and imaging,^23^ data on the correlation of liver stiffness improvement with histological fibrosis and cirrhosis regression remains lacking.^3^ Given the longer timeline required to demonstrate fibrosis improvement, trials should ideally be at least 1-2 years in duration.^47^

Kleiner et al demonstrated that a change of 10 U/L in liver enzymes (ALT and AST) was associated with a clinically significant change in liver fibrosis score in an adult population with biopsy-proven NAFLD, borderline NASH, and definitive NASH.^46^ Others have demonstrated that a 30% reduction in ALT is associated with improved histological features of NASH.^48,49^ The mean difference in ALT of 11.81 U/L may be clinically significant.

### Limitations

There were several limitations to this review. Only 3 of the 6 studies reported on our primary outcome of interest (resolution of NASH on biopsy); given the low number of patients, there is still only moderate certainty in the evidence. We had included adolescents in our inclusion criteria and unfortunately no studies have been completed in this population, though we note one RCT in patients aged 10-21 years is currently recruiting.^50^ In addition, the new MASLD terminology was introduced in June 2023; it is possible that some papers may have been published between then and our search date. Future updates to this systematic review should include the new terminology. Due to the heterogeneity in reported results, we used multiple imputations to derive SD for secondary outcomes, which may have impacted accuracy. Additional sources of bias were the lack of blinding in one study^40^ and another study used lifestyle restrictions without placebo treatment compared to a treatment group that did not undergo lifestyle changes.^39^ Finally, a range of doses using different GLP-1RA could lead to variability in results that could not be analyzed with sensitivity analysis due to low number of studies.

## Conclusions

Evidence is growing for the use of GLP-1RA in the treatment of NAFLD; this study adds to the literature in support of the potential histological resolution of NASH. However, larger prospective studies using histological outcomes and longer treatment duration are required to validate these results. The effects of GLP-1RA on improvement in fibrosis continue to be unclear and therefore treatment with GLP-1RA to specifically target NAFLD cannot be recommended. This review should be updated once more of the ongoing RCTs have reported their results.

## Supplementary data

Supplementary data are available at *Journal of the Canadian Association of Gastroenterology* online.

gwae057_suppl_Supplementary_Material

gwae057_suppl_Supplementary_ICMJE_Forms

## Data Availability

Data collection template form and extracted data can be made available upon request.
