# Supplementary material for: Effects of GLP-1 receptor agonist therapy on resolution of steatohepatitis in non-alcoholic fatty liver disease: a systematic review and meta-analysis
Source: J Can Assoc Gastroenterol. 2025 Jan 29;8(2):47–57. doi: 10.1093/jcag/gwae057 (PMC11991874; doi:10.1093/jcag/gwae057)
Supplement: gwae057_suppl_Supplementary_Material [file gwae057_suppl_supplementary_material.docx]

Supplemental Table 1: ROB assessment for primary outcome of each included study

1. Resolution of NASH on biopsy: Armstrong *et al.*

| Category for ROB | Authors’ judgment | Support for this decision |
| --- | --- | --- |
| ROB from randomization process | Low | No concerns |
| ROB from effect of assignment to intervention | Low | No concerns |
| ROB from missing outcome data | Low | No concerns |
| ROB from measurement of the outcome | Low | No concerns |
| ROB in selection of the reported result | Low | No concerns |
| Overall | Low | No concerns |

1. Change in liver stiffness on MRE: Flint *et al.*

| Category for ROB | Authors’ judgment | Support for this decision |
| --- | --- | --- |
| ROB from randomization process | Low | No concerns |
| ROB from effect of assignment to intervention | Low | No concerns |
| ROB from missing outcome data | Low | No concerns |
| ROB from measurement of the outcome | Some concerns | Participants were treated until 72 weeks but somewhat arbitrarily without explanation the outcomes at 48 weeks were chosen to be the primary outcome. No protocol was published ahead of time to demonstrate this was chosen a priori and may have been chosen on the basis of more favorable looking results at this time. |
| ROB in selection of the reported result | Low | No concerns |
| Overall | Some concerns | Some concerns in ROB in measurement of the data |

1. Change in intra-hepatic liver steatosis on MRI: Guo *et al.*

| Category for ROB | Authors’ judgment | Support for this decision |
| --- | --- | --- |
| ROB from randomization process | High ROB | No allocation concealment |
| ROB from effect of assignment to intervention | Some concerns | Study investigators, trial staff, and participants were very likely aware of who was in which study arm. There was no analysis to estimate the effect of assignment to treatment |
| ROB from missing outcome data | Low | No concerns |
| ROB from measurement of the outcome | Some concerns | Radiologists measuring H-MRS were unblinded, which introduces possible bias into their measurement of outcome data |
| ROB in selection of the reported result | High ROB | Unclear that data analysis was performed according to a pre-specified plan  Unclear if liver function was part of originally intended outcomes. They reported SAT and VAT as primary outcomes which were not specified in registry protocol |
| Overall | High ROB | High ROB in multiple domains |

1. Change in liver steatosis on MRE: Khoo *et al.*

| Category for ROB | Authors’ judgment | Support for this decision |
| --- | --- | --- |
| ROB from randomization process | Some concerns | There were some baseline differences between treatment and standard of care arms that would not be expected from randomization |
| ROB from effect of assignment to intervention | Some concerns | No intention to treat analysis specified |
| ROB from missing outcome data | Low | No concerns |
| ROB from measurement of the outcome | Low | No concerns |
| ROB in selection of the reported result | Low | No concerns |
| Overall | Some concerns | Some concerns in 2 domains |

1. Improvement in liver fibrosis on biopsy: Loomba *et al.*

| Category for ROB | Authors’ judgment | Support for this decision |
| --- | --- | --- |
| ROB from randomization process | Low | No concerns |
| ROB from effect of assignment to intervention | Low | No concerns |
| ROB from missing outcome data | Low | No concerns |
| ROB from measurement of the outcome | Low | No concerns |
| ROB in selection of the reported result | Low | No concerns |
| Overall | Low | No concerns |

1. Resolution of NASH on biopsy: Newsome *et al.*

| Category for ROB | Authors’ judgment | Support for this decision |
| --- | --- | --- |
| ROB from randomization process | Low | No concerns |
| ROB from effect of assignment to intervention | Low | No concerns |
| ROB from missing outcome data | Low | No concerns |
| ROB from measurement of the outcome | Low | No concerns |
| ROB in selection of the reported result | Low | No concerns |
| Overall | Low | No concerns |


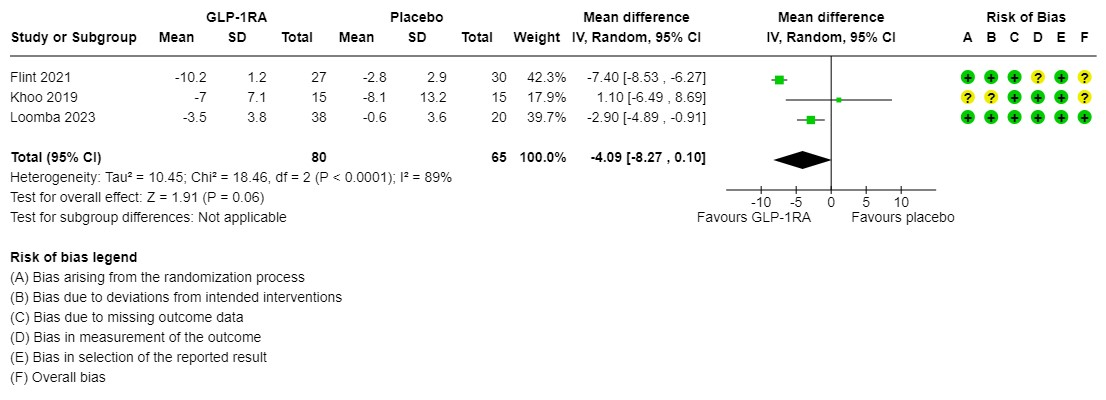
**Supplemental Figure 1. Sensitivity analysis for the effect of GLP-1RA on hepatic steatosis by MRI-PDFF(%) with exclusion of high risk of bias studies.** *For the Risk of Bias assessment, a green circle with a “+” sign symbolizes “low risk of bias,” a yellow circle with a “?” symbolizes “some concerns of potential bias,” and a red circle with a “-“ symbolizes “high risk of bias.”


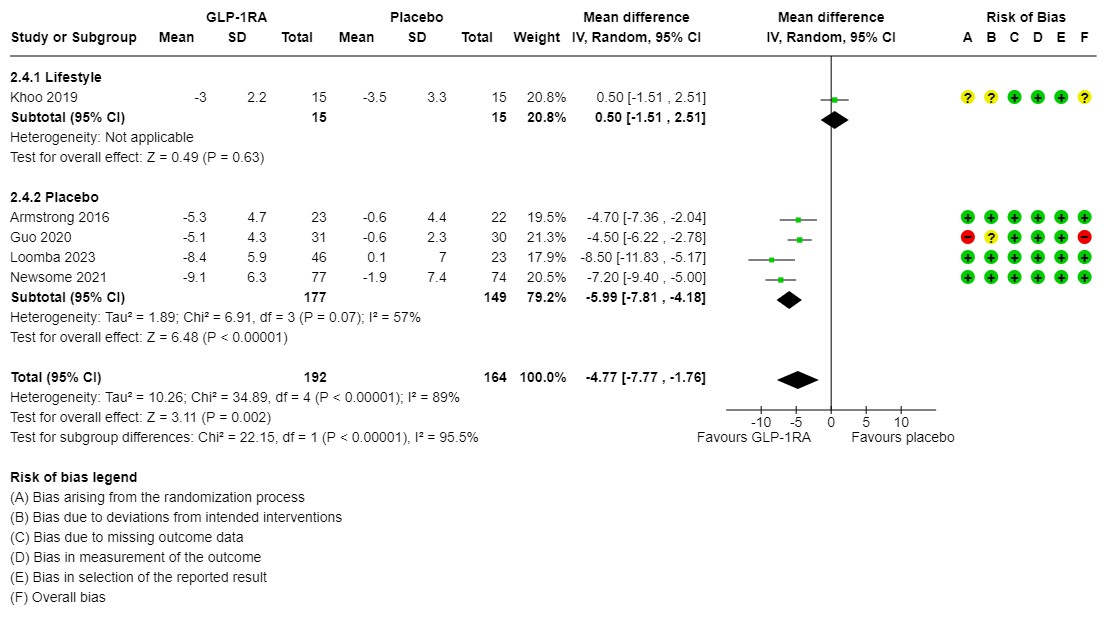
**Supplemental Figure 2. Effect of GLP-1RA on weight over 26 to 72 weeks** *For the Risk of Bias assessment, a green circle with a “+” sign symbolizes “low risk of bias,” a yellow circle with a “?” symbolizes “some concerns of potential bias,” and a red circle with a “-“ symbolizes “high risk of bias.”


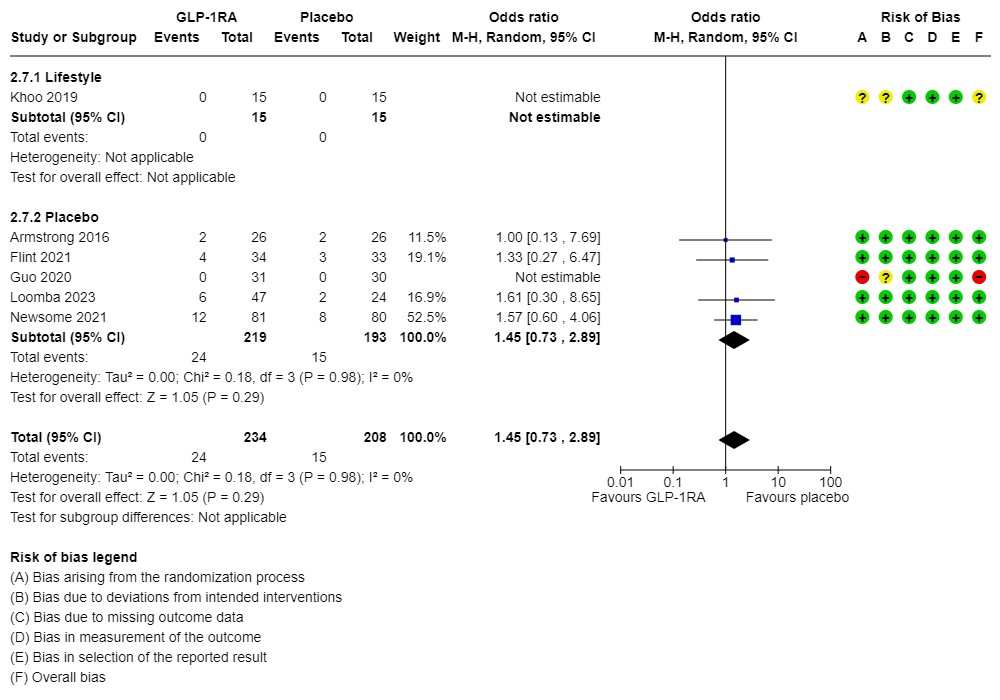

**Supplemental Figure 3. Effect of GLP-1RA on serious adverse events over 26-72 weeks.** *For the Risk of Bias assessment, a green circle with a “+” sign symbolizes “low risk of bias,” a yellow circle with a “?” symbolizes “some concerns of potential bias,” and a red circle with a “-“ symbolizes “high risk of bias.”

Appendix 1:

**Ovid MEDLINE(R) ALL <1946 to November 6, 2023>**

1. Non-alcoholic Fatty Liver Disease/ 24596
2. NAFL*.ti,ab,kf. 26221
3. NASH.ti,ab,kf. 13605
4. MAFL*.ti,ab,kf. 1478
5. MASH.ti,ab,kf. 1672
6. ((non-alcoholic or nonalcoholic or metabolic) adj4 (fatty liver* or steatohepatit*)).ti,ab,kf. 40459
7. 1 OR 2 OR 3 OR 4 OR 5 OR 6 48438
8. exp Glucagon-Like Peptide 1/ 11224
9. Exenatide/ 2912
10. Glucagon-Like Peptide-1 Receptor/ 4916
11. glucagon-like peptide-1.ti,ab,kf. 15847
12. glp-1.ti,ab,kf. 15100
13. glp-1RA.ti,ab,kf. 884
14. exenatide.ti,ab,kf. 2363
15. albiglutide.ti,ab,kf. 243
16. liraglutide.ti,ab,kf. 3925
17. semaglutide.ti,ab,kf. 1318
18. dulaglutide.ti,ab,kf. 723
19. lixisenatide.ti,ab,kf. 567
20. 8 OR 9 OR 10 OR 11 OR 12 OR 13 OR 14 OR 15 OR 16 OR 17 OR 18 OR 19 24737
21. 7 AND 20 777
22. Exp randomized controlled trial/ 603994
23. controlled clinical trial.pt. 95445
24. randomized.ab. 623169
25. placebo.ab. 242860
26. drug therapy.fs. 2636174
27. randomly.ab. 420084
28. trial.ab. 671046
29. groups.ab. 2591815
30. 22 OR 23 OR 24 OR 25 OR 26 OR 27 OR 28 OR 29 5795390
31. Exp animals/ NOT humans.sh. 5167250
32. 30 NOT 31 5062741
33. 21 AND 32 **392 Results**

**Embase (Elsevier; 1974 to November 6, 2023)**

1. 'nonalcoholic fatty liver'/exp 70577
2. nafl*:ti,ab,kw 44,887
3. nash:ti,ab,kw 26740
4. mafl*:ti,ab,kw 1973
5. mash:ti,ab,kw 2007
6. ((non-alcoholic or nonalcoholic or metabolic) NEAR/4 (‘fatty liver*’ or steatohepatit*)):ti,ab,kw 64326
7. #1 OR #2 OR #3 OR #4 OR #5 OR #6 89567
8. 'glucagon like peptide 1'/de 24288
9. ‘exendin 4’/de 12289
10. ‘glucagon like peptide 1 receptor’/de 5839
11. ‘glucagon-like peptide-1’:ti,ab,kw 21877
12. 'glp-1':ti,ab,kw 25238
13. 'glucagon like peptide 1 receptor agonist'/exp 49099
14. ‘glp-1ra’:ti,ab,kw 1527
15. exenatide:ti,ab,kw 4496
16. albiglutide:ti,ab,kw 425
17. liraglutide:ti,ab,kw 7447
18. semaglutide:ti,ab,kw 2417
19. dulaglutide:ti,ab,kw 1424
20. lixisenatide:ti,ab,kw 975
21. #8 OR #9 OR #10 OR #11 OR #12 OR #13 OR #14 OR #15 OR #16 OR #17 OR #18 OR #19 OR #20 54030
22. #7 and #21 2628
23. ‘randomized controlled trial’/exp 792993
24. ‘controlled clinical trial’/de 441152
25. random*:ti,ab,tt 1987622
26. ‘randomization’/de 98676
27. ‘intermethod comparison’/de 303809
28. placebo:ti,ab,tt 367790
29. compare:ti,tt OR compared:ti,tt OR comparison:ti,tt 631186
30. ((evaluated:ab OR evaluate:ab OR evaluating:ab OR assessed:ab OR assess:ab) AND (compare:ab OR compared:ab OR comparing:ab OR comparison:ab)) 2798538
31. (open NEXT/1 label):ti,ab,tt 109588
32. ((double OR single OR doubly OR singly) NEXT/1 (blind OR blinded OR blindly)):ti,ab,tt 276977
33. ‘double blind procedure’/de 212217
34. (parallel NEXT/1 group*):ti,ab,tt 32381
35. crossover:ti,ab,tt OR ‘cross over’:ti,ab,tt 125351
36. ((assign* OR match OR matched OR allocation) NEAR/6 (alternate OR group OR groups OR intervention OR interventions OR patient OR patients OR subject OR subjects OR participant OR participants)):ti,ab,tt 460656
37. assigned:ti,ab,tt OR allocated:ti,ab,tt 493349
38. (controlled NEAR/8 (study OR design OR trial)):ti,ab,tt 462783
39. volunteer:ti,ab,tt OR volunteers:ti,ab,tt 285118
40. ‘human experiment’/de 651490
41. trial:ti,tt 411143
42. #23 OR #24 OR #25 OR #26 OR #27 OR #28 OR #29 OR #30 OR #31 OR #32 OR #33 OR #34 OR #35 OR #36 OR #37 OR #38 OR #39 OR #40 OR #41 6417201
43. ((random* NEXT/1 sampl* NEAR/8 (‘cross section*’ OR questionnaire* OR survey OR surveys OR database or databases)):ti,ab,tt) NOT (‘comparative study’/de OR ‘controlled study’/de OR ‘randomised controlled’:ti,ab,tt OR ‘randomized controlled’:ti,ab,tt OR ‘randomly assigned’:ti,ab,tt) 3167
44. ‘cross-sectional study’/de NOT (‘randomized controlled trial’/exp OR ‘controlled clinical study’/de OR ‘controlled study’/de OR ‘randomised controlled’:ti,ab,tt OR ‘randomized controlled’:ti,ab,tt OR ‘control group’:ti,ab,tt OR ‘control groups’:ti,ab,tt) 362956
45. ‘case control*’:ti,ab,tt AND random*:ti,ab,tt NOT (‘randomised controlled’:ti,ab,tt OR ‘randomized controlled’:ti,ab,tt) 21607
46. ‘systematic review’:ti,tt NOT (trial:ti,tt OR study:ti,tt) 262983
47. nonrandom*:ti,ab,tt NOT random*:ti,ab,tt 19002
48. ‘random field*’:ti,ab,tt 2933
49. (‘random cluster’ NEAR/4 sampl*):ti,ab,tt 1601
50. review:ab AND review:it NOT trial:ti,tt 1127471
51. ‘we searched’:ab AND (review:ti,tt OR review:it) 49756
52. ‘update review’:ab 137
53. (databases NEAR/5 searched):ab 67781
54. (rat:ti,tt OR rats:ti,tt OR mouse:ti,tt OR mice:ti,tt OR swine:ti,tt OR porcine:ti,tt OR murine:ti,tt OR sheep:ti,tt OR lambs:ti,tt OR pigs:ti,tt OR piglets:ti,tt OR rabbit:ti,tt OR rabbits:ti,tt OR cat:ti,tt OR cats:ti,tt OR dog:ti,tt OR dogs:ti,tt OR cattle:ti,tt OR bovine:ti,tt OR monkey:ti,tt OR monkeys:ti,tt OR trout:ti,tt OR marmoset*:ti,tt) AND ‘animal experiment’/de 1230671
55. ‘animal experiment’/de NOT (‘human experiment’/de OR ‘human’/de) 2584601
56. #43 OR #44 OR #45 OR #46 OR #47 OR #48 OR #49 OR #50 OR #51 OR #52 OR #53 OR #54 OR #55 4365437
57. #42 NOT #56 5669609
58. #22 AND #57 **493 Results**

RCT search filters for MEDLINE (Ovid) and Embase (Elsevier) taken from the Cochrane Handbook:

Lefebvre C, Glanville J, Briscoe S, Featherstone R, Littlewood A, Metzendorf M-I, Noel-Storr A, Paynter R, Rader T, Thomas J, Wieland LS. Chapter 4: Searching for and selecting studies. In: Higgins JPT, Thomas J, Chandler J, Cumpston M, Li T, Page MJ, Welch VA (editors). Cochrane Handbook for Systematic Reviews of Interventions version 6.4 (updated October 2023). Cochrane, 2023. Available from www.training.cochrane.org/handbook.

**Cochrane Central Register of Controlled Trials CENTRAL (Inception to November 6, 2023)**

1. [mh ^”Non-alcoholic Fatty Liver Disease”] 1642
2. NAFL*:ti,ab,kw 2733
3. NASH:ti,ab,kw 1769
4. MAFL*:ti,ab,kw 90
5. MASH:ti,ab,kw 79
6. ((non-alcoholic OR nonalcoholic OR metabolic) near/4 ((fatty NEXT liver*) OR steatohepatit*)):ti,ab,kw 4721
7. #1 OR #2 OR #3 OR #4 OR #5 OR #6 5201
8. [mh "Glucagon-Like Peptide 1"] 2196
9. [mh ^”Exenatide”] 628
10. [mh ^”Glucagon-Like Peptide-1 Receptor”] 326
11. "glucagon-like peptide-1":ti,ab,kw 4238
12. GLP-1:ti,ab,kw 4202
13. GLP-1RA:ti,ab,kw 287
14. exenatide:ti,ab,kw 1322
15. albiglutide:ti,ab,kw 139
16. liraglutide:ti,ab,kw 2297
17. semaglutide:ti,ab,kw 1025
18. dulaglutide:ti,ab,kw 533
19. lixisenatide:ti,ab,kw 364
20. #8 OR #9 OR #10 OR #11 OR #12 OR #13 OR #14 OR #15 OR #16 OR #17 OR #18 OR #19 8637
21. #7 AND #20 **233 Results**

ClinicalTrials.gov (November 16, 2023)

*Condition/disease:*

"Non-Alcoholic Fatty Liver" OR “Metabolic Fatty Liver” OR NAFL OR NASH OR MAFL OR MASH OR Steatohepatitis

AND

*Intervention/treatment:*

Glucagon OR Exenatide OR GLP OR GLP-1 OR Albiglutide OR Liraglutide OR Semaglutide OR Dulaglutide OR Lixisenatide

*Study status: All studies*

**60 Results**

International Clinical Trials Registry Platform (ICTRP) (Nov 16, 2023)

("Non-Alcoholic Fatty Liver" OR “Metabolic Fatty Liver” OR NAFL OR NASH OR MAFL OR MASH OR Steatohepatitis) AND (Glucagon OR Exenatide OR GLP OR GLP-1 OR Albiglutide OR Liraglutide OR Semaglutide OR Dulaglutide OR Lixisenatide)

*No filters used*

**107 records for 69 trials (NOTE: 69 records were exported due to the portal automatically batching duplicate records into a single record)**
